# Supplementary material for: Breath biomarkers of insulin resistance in pre-diabetic Hispanic adolescents with obesity
Source: Sci Rep. 2022 Jan 10;12:339. doi: 10.1038/s41598-021-04072-3 (PMC8748903; doi:10.1038/s41598-021-04072-3)
Supplement: Supplementary file 1 — Supplementary Information. [file 41598_2021_4072_MOESM1_ESM.docx]

**Supplementary File**

**Breath biomarkers of insulin resistance in pre-diabetic Hispanic adolescents with obesity**

*Mohammad S. Khan,^1,2^ PhD, Suzanne Cuda^3,4^ MD, Genesio M. Karere^1,2^ PhD, Laura Cox^1,2^ PhD, and Andrew C. Bishop^1,2^ PhD*^*^

^1^Department of Internal Medicine, Section on Molecular Medicine, Wake Forest School of Medicine, Winston Salem, NC 27157

^2^Center for Precision Medicine. Wake Forest School of Medicine, Winston Salem, NC 27157

^3^Health and Weight Management Clinic, Children’s Hospital of San Antonio, San Antonio, TX 78207

^4^Baylor College of Medicine, Houston, TX 77030

*Corresponding author

Andrew C. Bishop, Ph.D.

Department of Internal Medicine, Section on Molecular Medicine

Wake Forest School of Medicine, Winston-Salem, NC

E: [abishop@wakehealth.edu](mailto:abishop@wakehealth.edu)

O: 336-713-7148


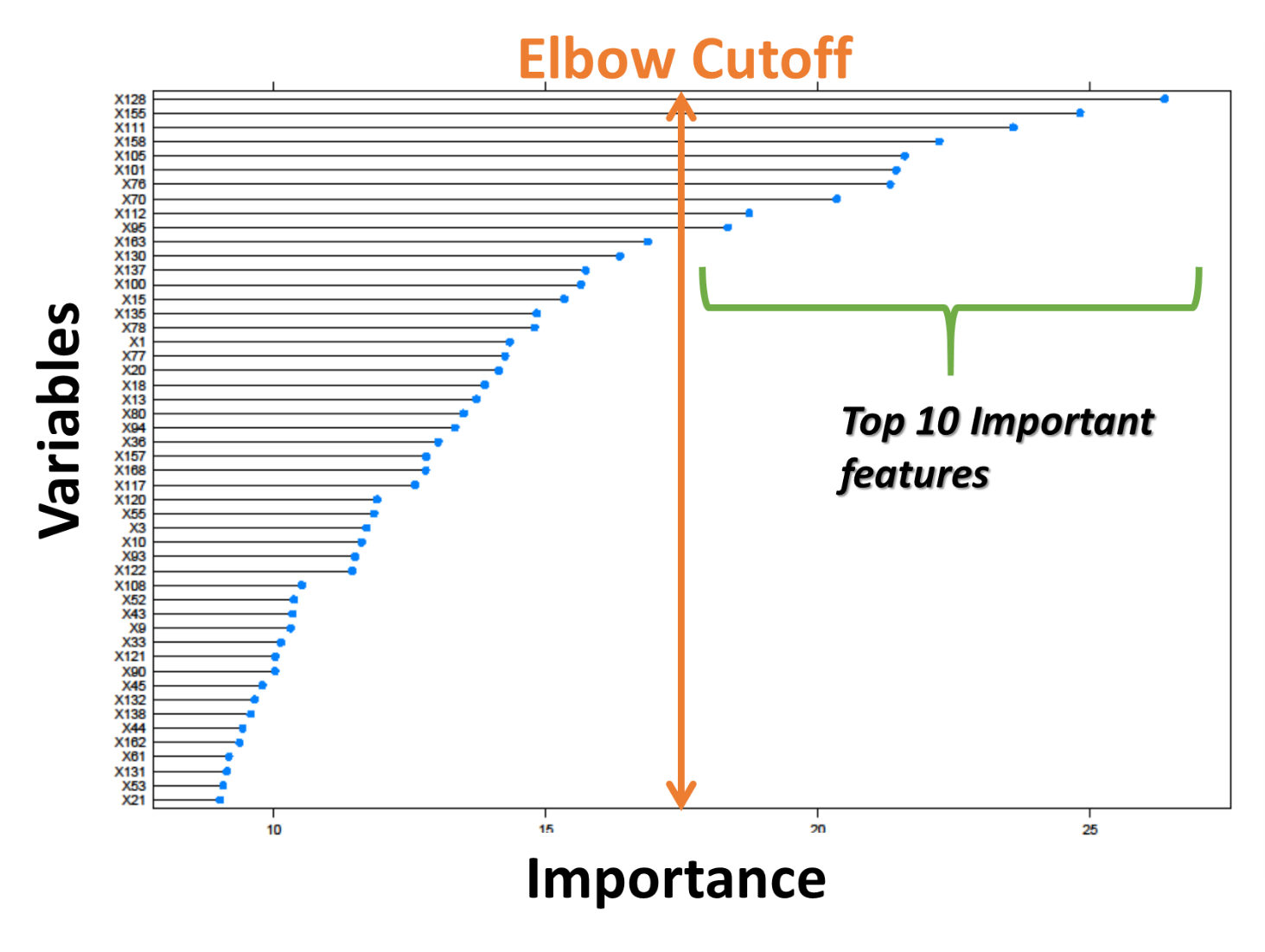


**Figure S1:** The importance plot of the Random Forest based feature selection. All variables are ranked based on the mean decease accuracy measure of Random forest. The elbow cutoff is based on the drop of importance which selected the top 10 important features for further analysis. The plot is generated in R^1^ using the ‘caret’^2^ package.

.

**Table S1:** Chromatographic and mass spectral identification of the ten compounds.

| **Features** | **FINAL ID** | **Formula** | **RI^a^** | **Chemical Class** | **^1^t_R_(s)** | **^1^t_R_(s)**  **(RSD %)** | **^2^t_R_(s)** | **^2^t_R_(s)**  **(RSD %)** | **ID**  **Matching^b^** |
| --- | --- | --- | --- | --- | --- | --- | --- | --- | --- |
| 70 | Unknown 1 |  |  |  | 564.3 | 0.3 | 0.2 | 30.0 |  |
| 76 | Unknown 2 |  |  |  | 594.3 | 0.3 | 1.3 | 1.9 |  |
| 95 | Limonene | C_10_H_16_ | 1063 | cyclic monoterpene | 693.3 | 0.3 | 1.8 | 1.4 | RI + MS |
| 101 | Decane, 2,4,6-trimethyl- | C_13_H_28_ | 1095 | branched hydrocarbon | 745.0 | 0.0 | 1.4 | 1.6 | RI + MS |
| 105 | Undecane | C_11_H_24_ | 1141 | hydrocarbon | 824.4 | 0.2 | 1.4 | 1.6 | RI + MS |
| 111 | Undecane 2,7 dimethyl | C_13_H_28_ | 1201 | hydrocarbon | 915.2 | 0.1 | 1.4 | 1.7 | RI + MS |
| 112 | pentylbenzene | C_11_H_16_ | 1205 | aromatic hydrocarbon | 920.0 | 0.0 | 2.0 | 1.7 | RI + MS |
| 128 | Octamethyloctane | C_16_H_34_ | 1396 | branched hydrocarbon | 1153.2 | 0.6 | 1.4 | 1.9 | RI + MS |
| 155 | Unknown 3 |  |  |  | 1563.8 | 0.1 | 1.5 | 3.3 |  |
| 158 | Eicosane | C_20_H_42_ | 1938 | hydrocarbon | 1618.8 | 0.1 | 1.6 | 2.3 | RI + MS |

^a^ Retention index was determined using C_8_ ~ C_22_ n‐alkane standard solution.

^b^ ID was matched by reported retention indices and mass-spectral matchings.


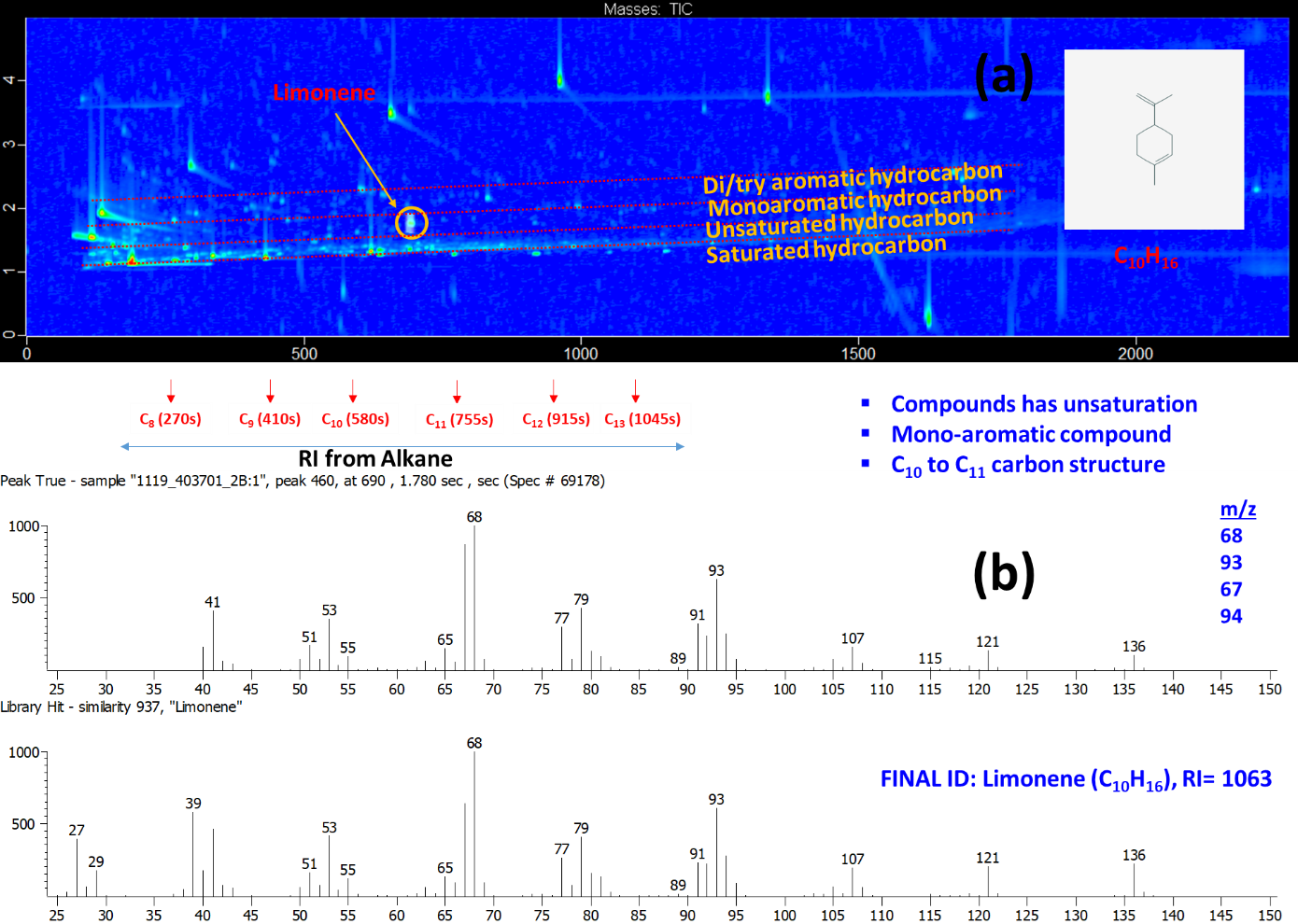


**Figure S2:** The example of a peak detection and identification used in this study. (a) The GC×GC contour plot of a breath sample. The group type separation indicate the peak is an unsaturated, mono terpene compounds. The RI of the peak indicate the position of the peak between C_10_ to C_11_. (b) The peak is then compared with the NIST library by m/z 68, 93, 67 and 94. Finally, combining all these info, a final ID was given as “limonene”. The GC×GC plot is generated in Chromatof software.

**
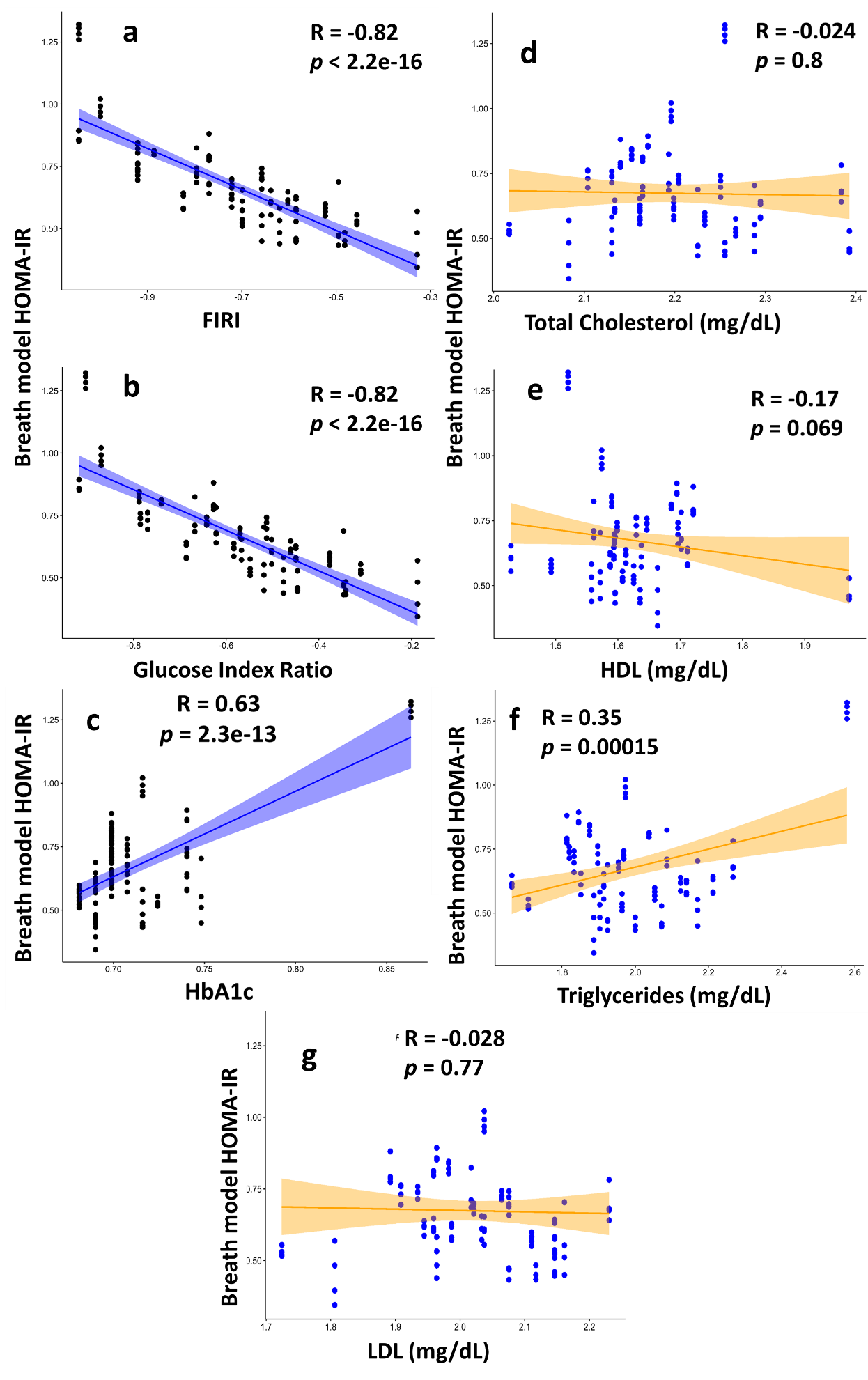
**

**Figure S3:** Correlation of breath based HOMA-IR model with Fasting insulin resistance index (a) (FIRI), (b) Glucose Insulin Ratio, (c) HbA1c, (d) Total cholesterol (mg/dL), (e) HDL (mg/dL), (f) Triglycerides (mg/dL), and (g) LDL (mg/dL). Data were log_10_-transformed. Pearson correlation was conducted in R^1^ using ‘ggpubr’^3^.


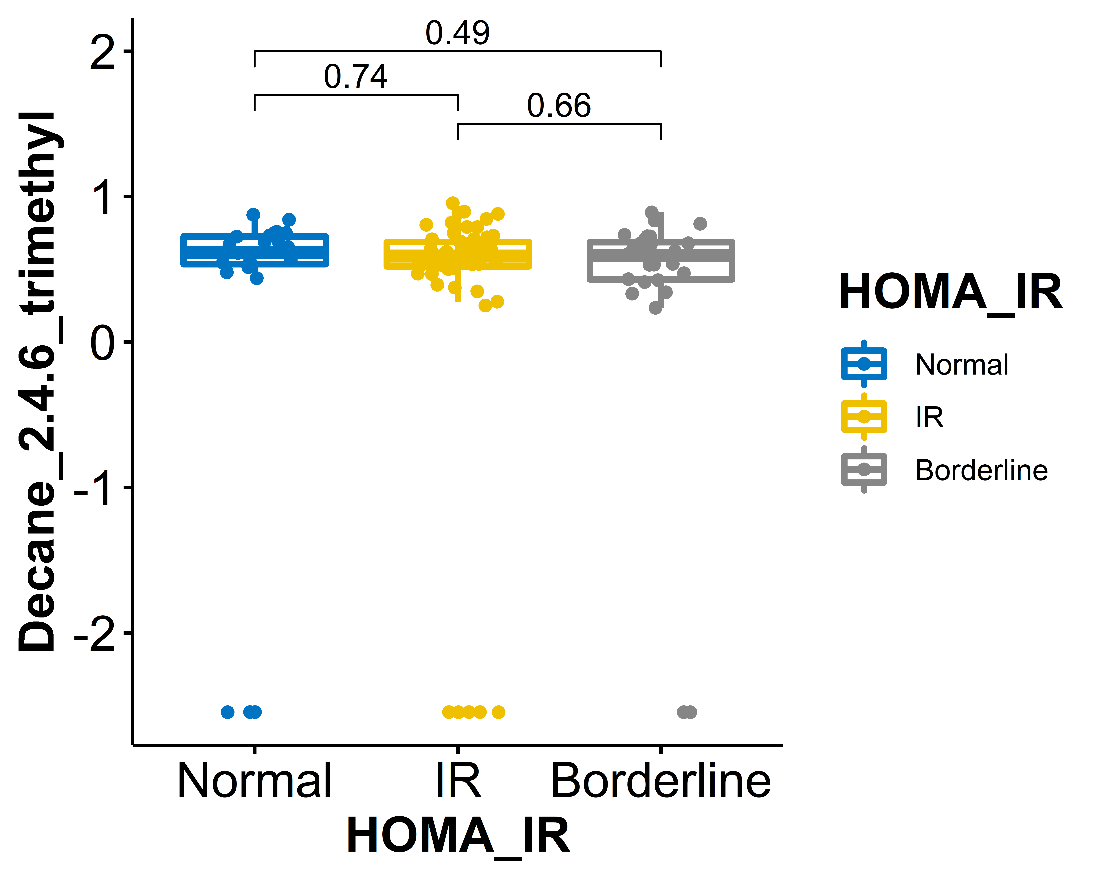

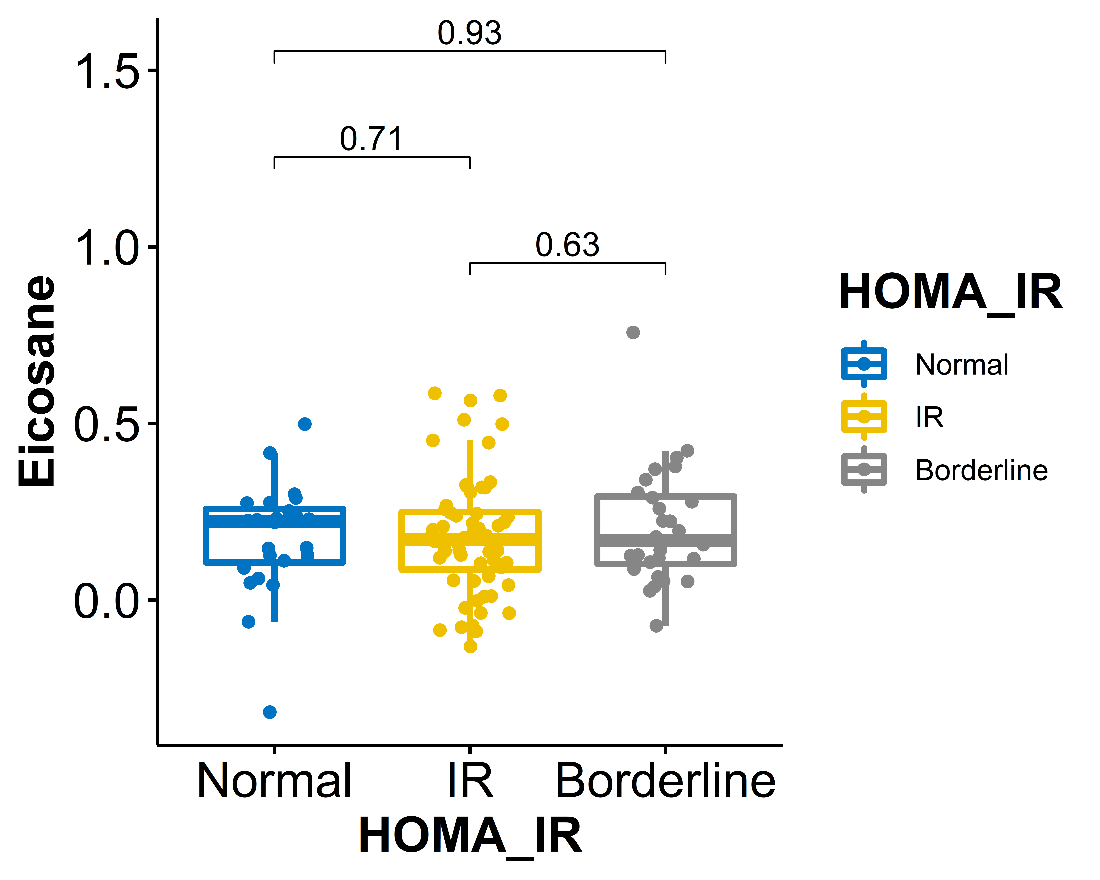

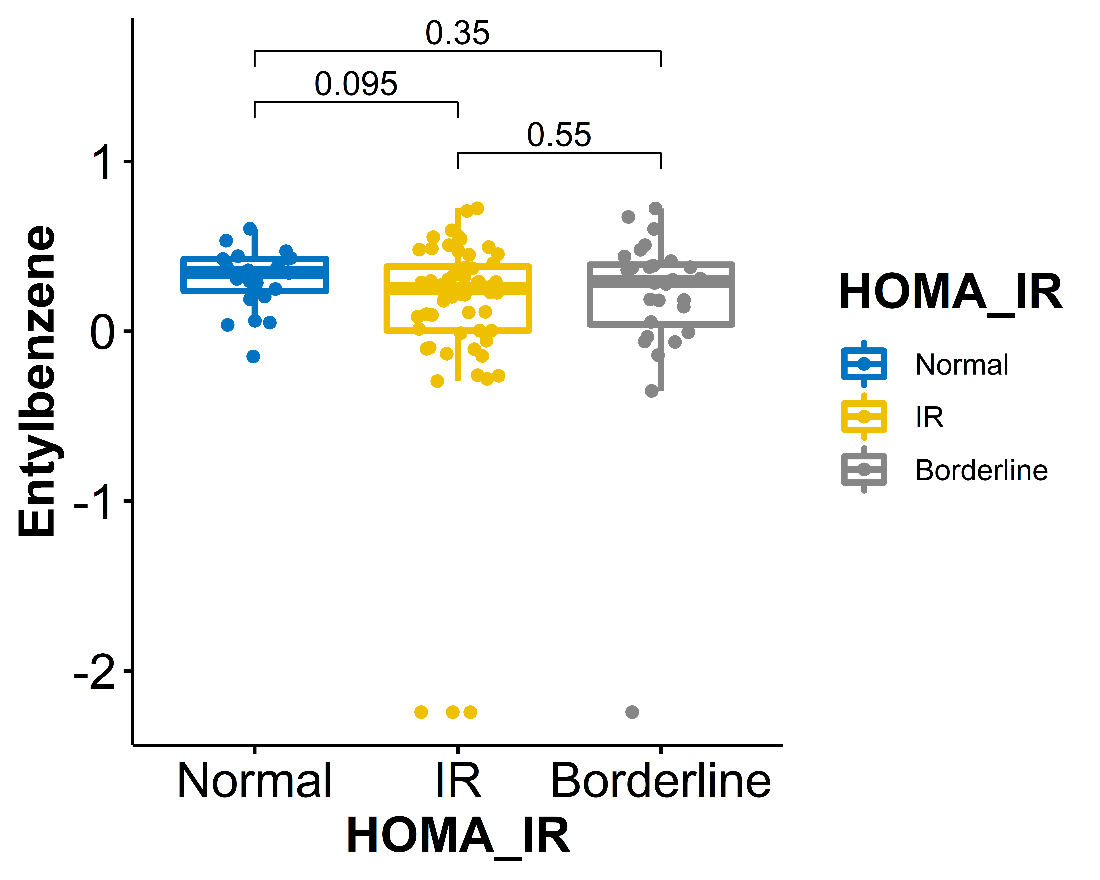

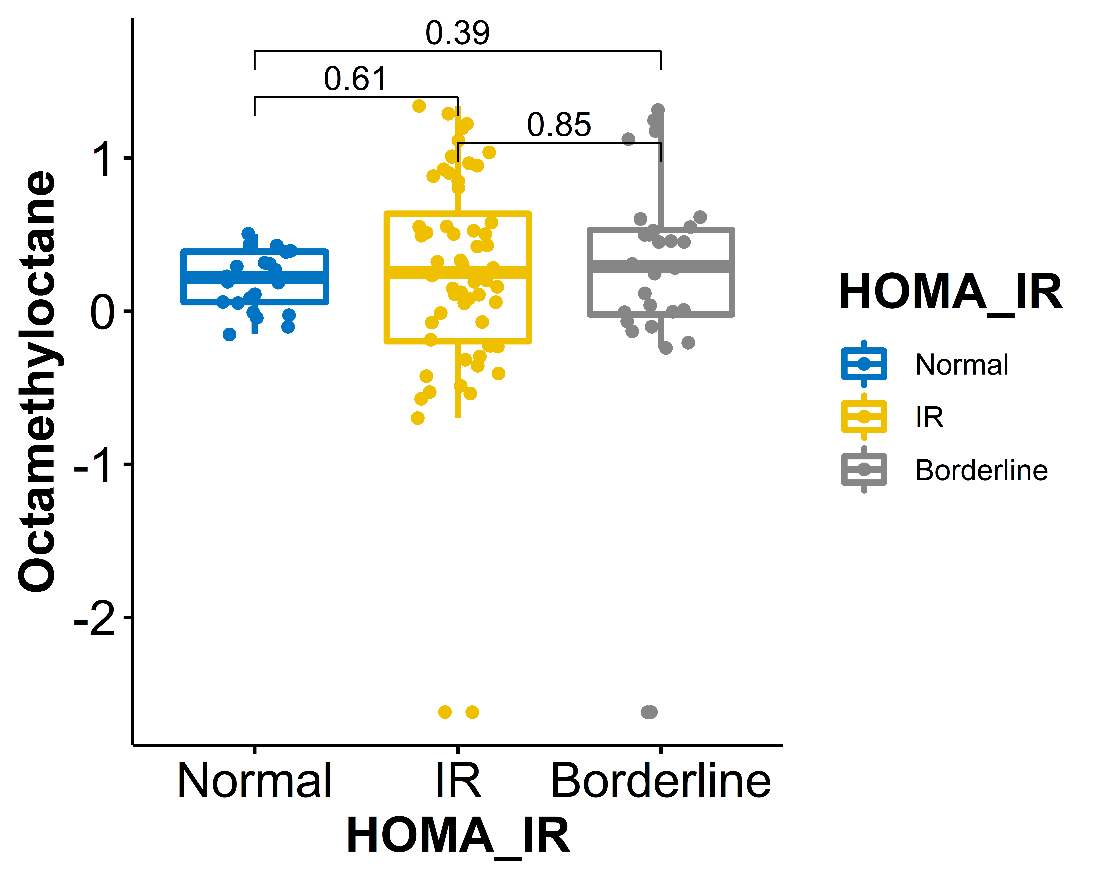

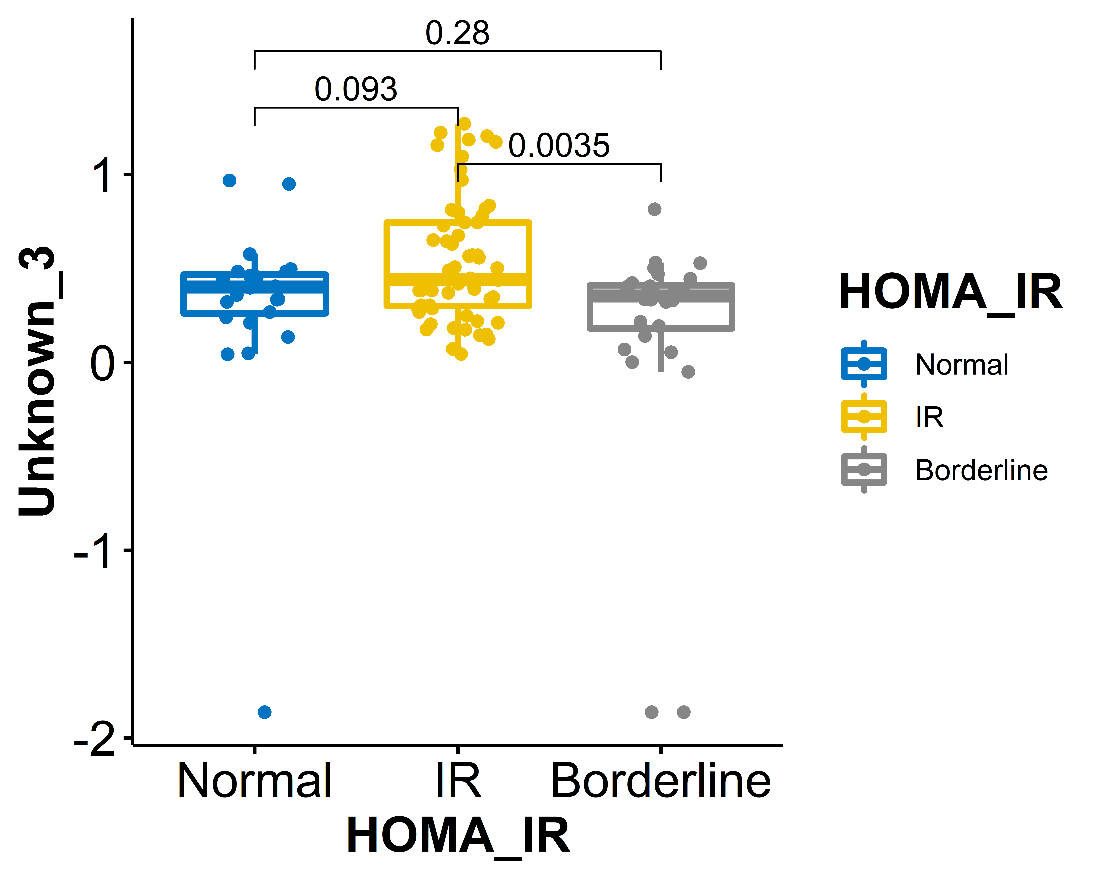


**a**

**b**

**c**

**d**

**g**


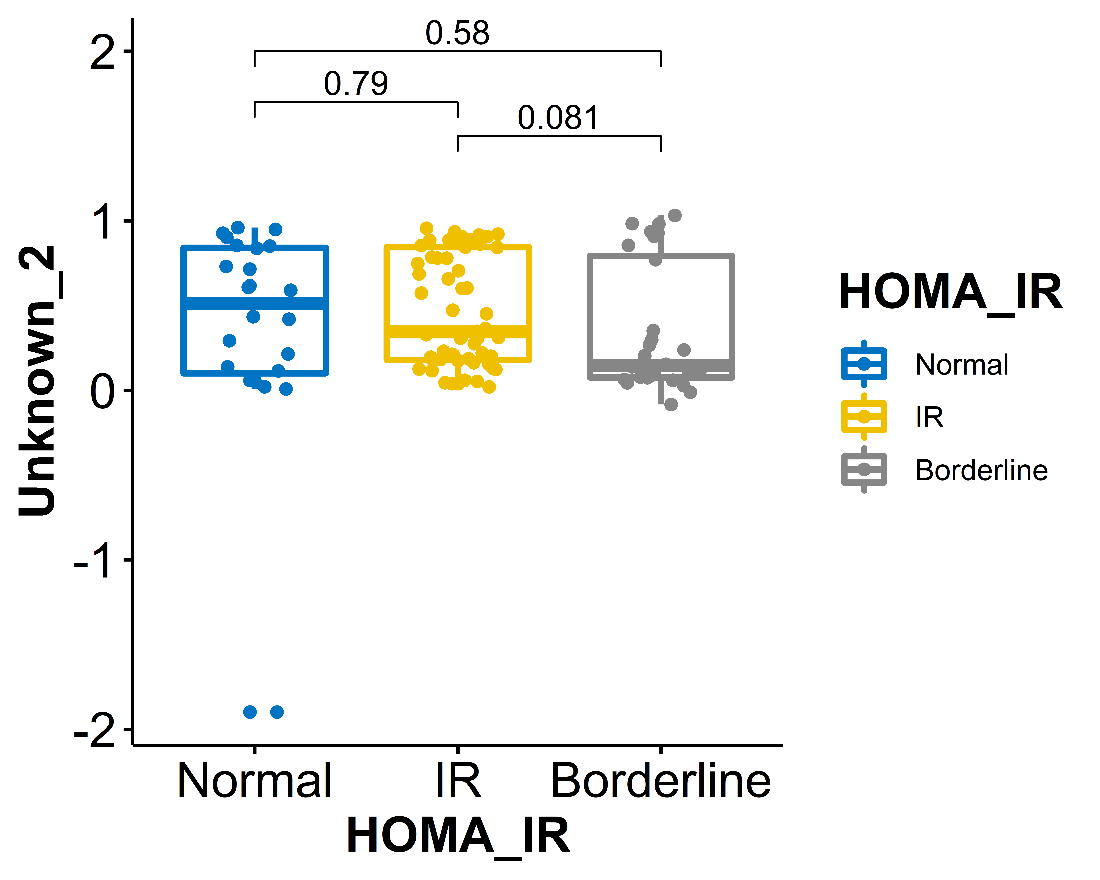

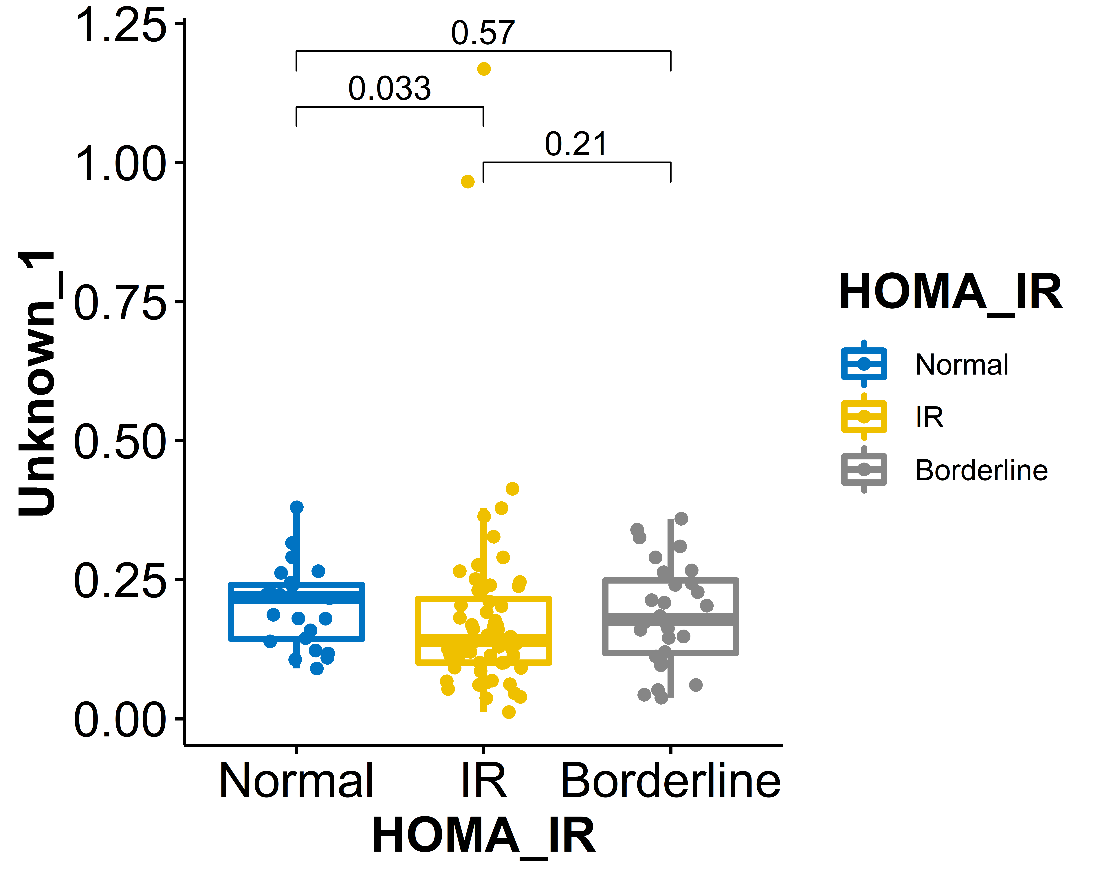


**g**

**f**

**e**

**
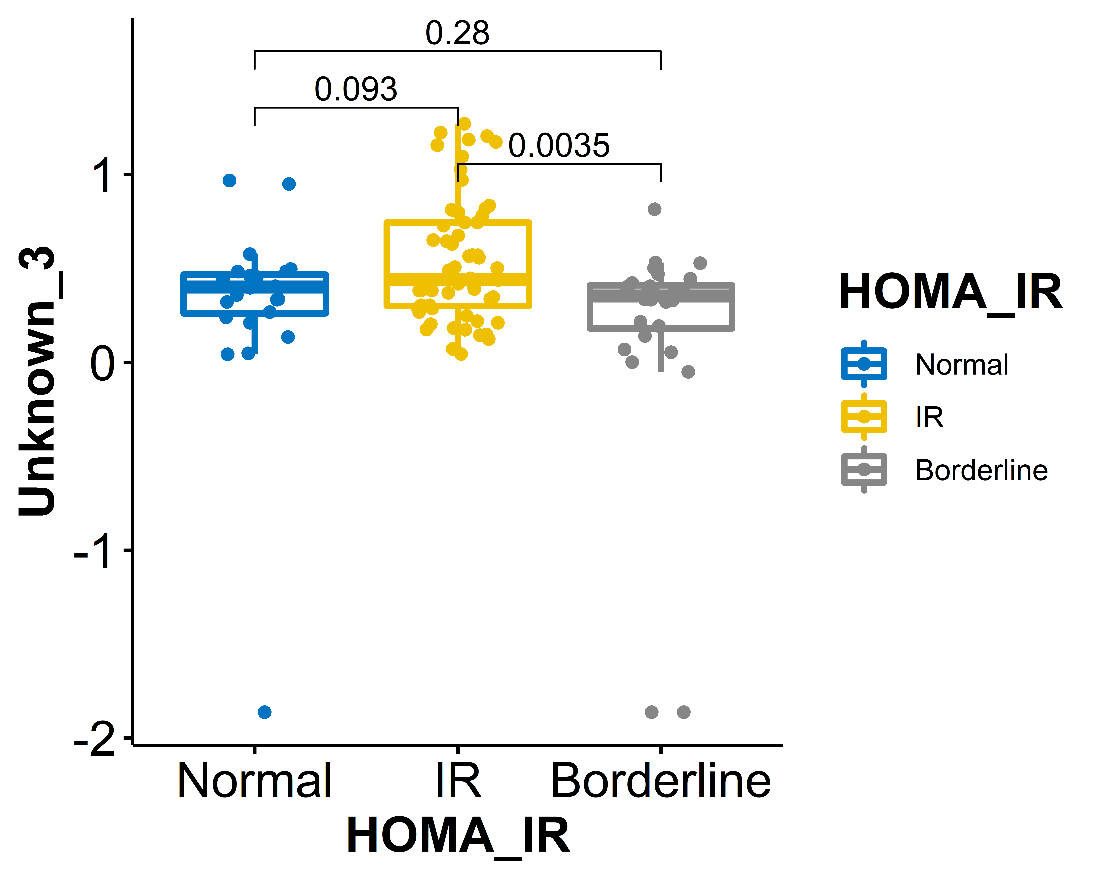
**

**Figure S4:** The boxplot of the mean centered and normalized peak area of (a) Decane_2.4.6_trimethyl, (b) Entylbenzene, (c) Eicosane, (d) Octamethyloctane, (e) Unknown_1 (f) Unknown_2, and (g) Unknown_3. Boxplots show the quartiles of the data (first line is the first quartile, midline is the median, third line is the third quartile) where whiskers represent 1.5 × IQR (inter-quartile range). The Wilcoxon non-parametric test was performed comparing each IR group type. Figure created in R^1^ using ‘ggplot2’^4^ and ‘ggpubr’^3^.

**
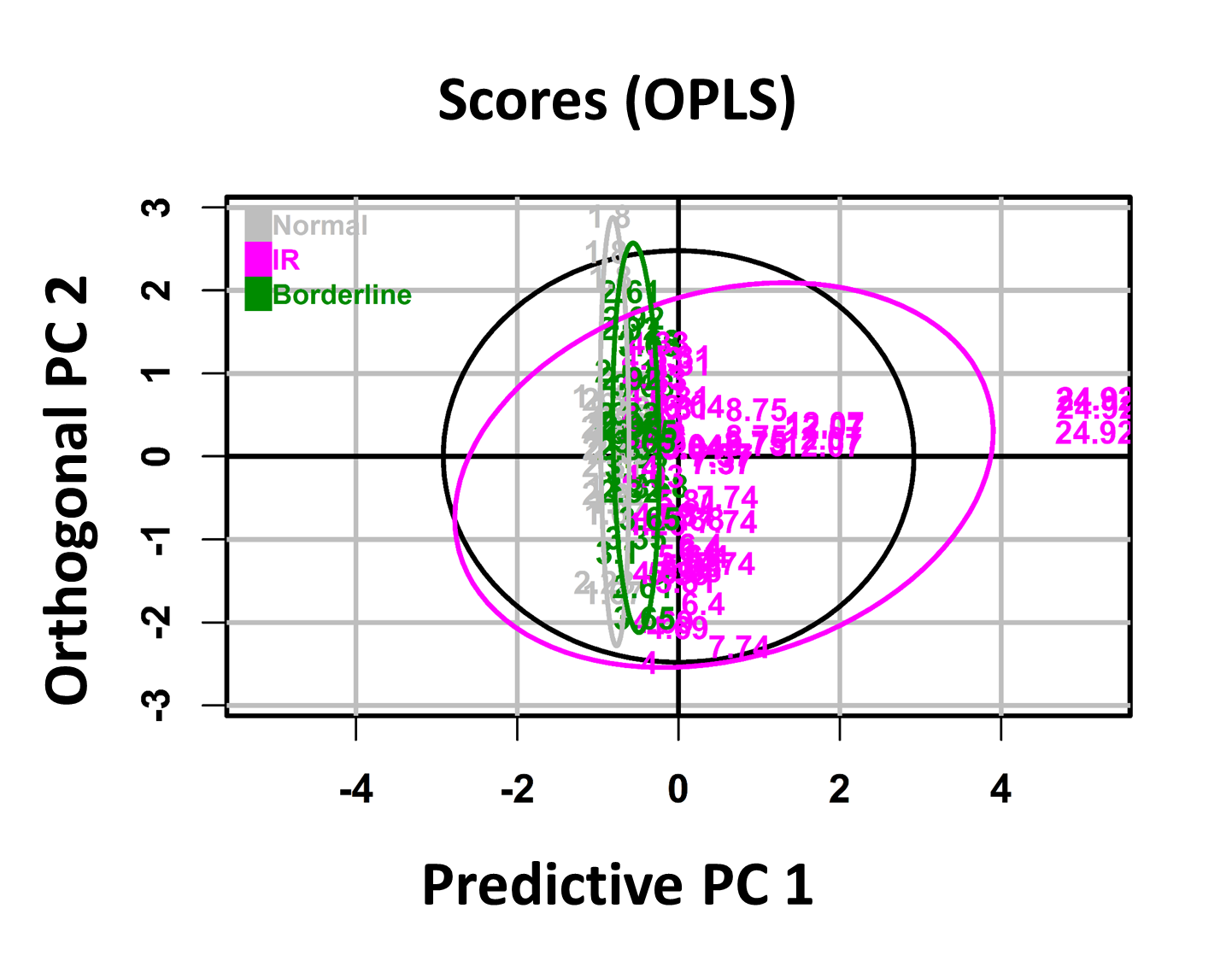
**

**Figure S5**: The orthogonal partial least-squares discriminant analysis (OPLS) plot of the IR samples with two extreme values. The purple color indicates the IR group with HOMA-IR>3.80, the green color represents the borderline group with HOMA-IR 2.60–3.80 and grey color indicates HOMA-IR<2.60 normal group. The 95% tolerance region corresponds to the ellipse that is defined by the Hotelling's T2 parameter. The OPLS analysis and figure created in R^1^ using ‘ropls’^5^.

**
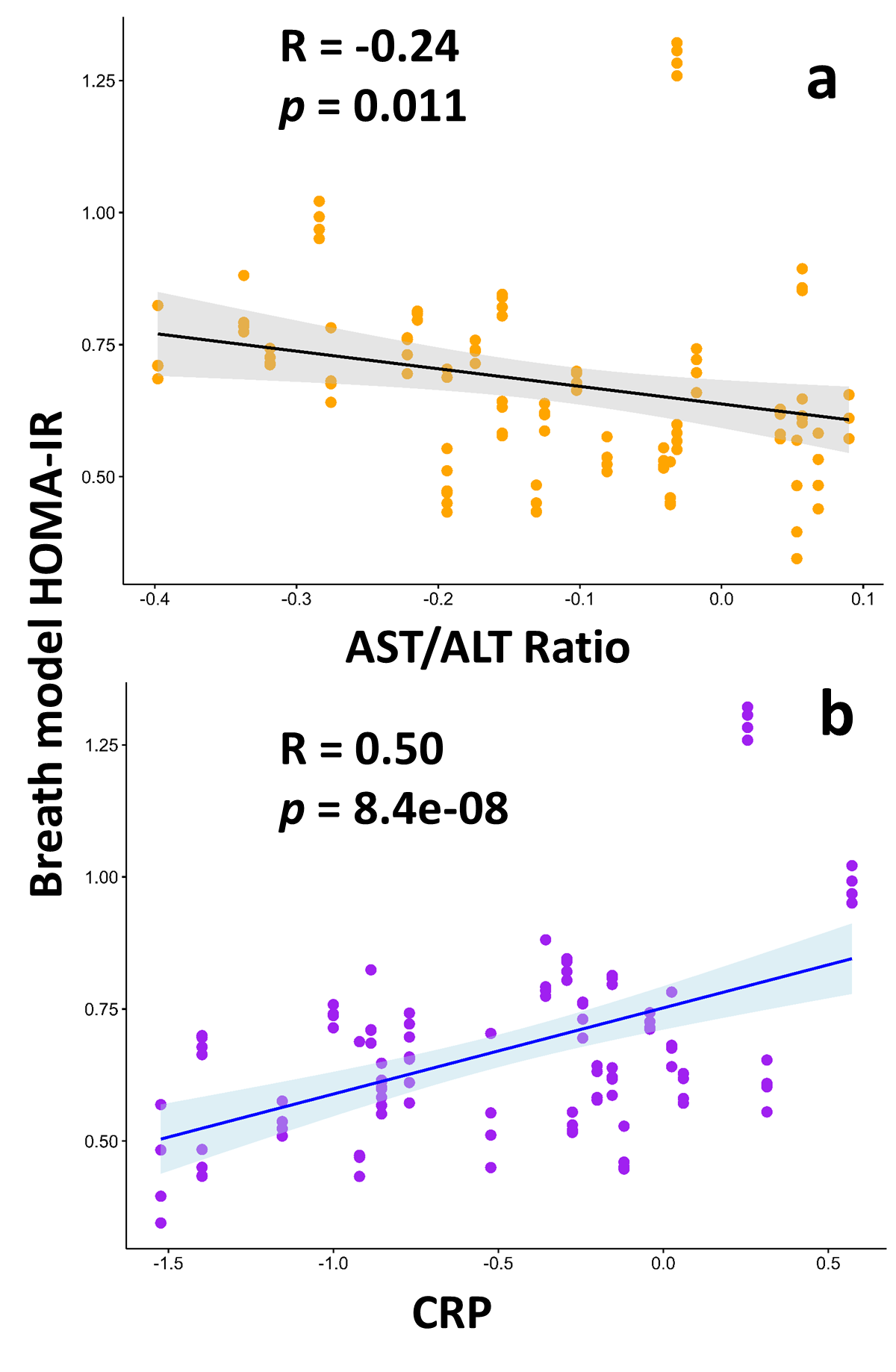
**

**Figure S6**: The correlation of the HOMA-IR of the adolescent, (a) the AST/ALT ratio and (b) c-reactive protein (CRP). Data were log_10_ -transformed. Pearson correlation was conducted in R^1^ using ‘ggpubr’^3^.

**Table S2:** List of known artifacts from the instrument and laboratory supplies used for the breath analysis are removed from the data analysis.

| **Contaminants Name** | **Frequency** |
| --- | --- |
| (2-(4-Isopropylphenyl)propan-2-ylperoxy)trimethylsilane | 2 |
| 1,1,1,3,5,5,5-Heptamethyltrisiloxane | 10 |
| 1,3,6-Trioxa-2-silacyclooctane, 2,2,-dimethylsilyl- | 2 |
| 1,3-Bis[methyl(trimethylene)silyloxy]propane | 1 |
| 1,3-Diallyl(tetramethyl)disiloxane | 1 |
| 1,3-Dioxa-2,4,6-trisilacyclohexane, 2,2,4,4,6,6-hexamethyl- | 1 |
| 1,4-Cyclohexadiene, 1,3,6-tris(trimethylsilyl)- | 1 |
| 2-Dimethyl(ethenyl)silyloxytetradecane | 1 |
| 2-Oxa-1,3-disilacyclohexane, 1,1,3,3-tetramethyl- | 1 |
| 4-Methyl-1-di(tert-butyl)silyloxypentane | 1 |
| Arsenous acid, tris(trimethylsilyl) ester | 1 |
| Carbamic acid, monoammonium salt | 17 |
| Carbon dioxide | 4 |
| Cyclotrisiloxane, hexamethyl- | 13 |
| Disiloxane, 1,3-diethyl-1,1,3,3,-tetramethyl- | 1 |
| Disiloxane, 1-ethenyl-1,1,3,3-tetramethyl-3-(2-propenyl)- | 4 |
| Disiloxane, ethylpentamethyl- | 4 |
| Disiloxane, hexamethyl- | 5 |
| Disiloxane, pentamethyl-2-propenyl- | 1 |
| Ethyl(dimethyl)isopropoxysilane | 1 |
| Methylene chloride | 2 |
| Nitrous oxide | 60 |
| Oxalic acid, 2TMS derivative | 4 |
| Oxalic acid, 6-ethyloct-3-yl isohexyl ester | 1 |
| Oxalic acid, butyl isobutyl ester | 1 |
| Oxalic acid, isobutyl nonyl ester | 1 |
| Oxalic acid, isobutyl pentyl ester | 1 |
| Phthalic acid, hex-3-yl isobutyl ester | 1 |
| Phthalic anhydride | 1 |
| Silane, diethoxydimethyl- | 1 |
| Silane, ethoxytriethyl- | 1 |
| Silane, methyldiisopropoxymethoxy- | 1 |
| Silane, methyltriisopropoxy- | 1 |
| Silane, tetramethyl- | 21 |
| Silane, trichlorodocosyl- | 1 |
| Silane, trimethyl-2-propenyl- | 1 |
| Silanediol, dimethyl-, diacetate | 1 |
| Silanol, trimethyl- | 4 |
| tert-Butyldimethylsilanol | 1 |
| tert-Butylpentamethyldisiloxane | 1 |
| Trimethyl(3,3-difluoro-2-propenyl)silane | 1 |
| Trimethylsilyl ethaneperoxoate | 1 |
| Trimethylsilyl-di(timethylsiloxy)-silane | 1 |
| Trisiloxane, octamethyl- | 12 |

**
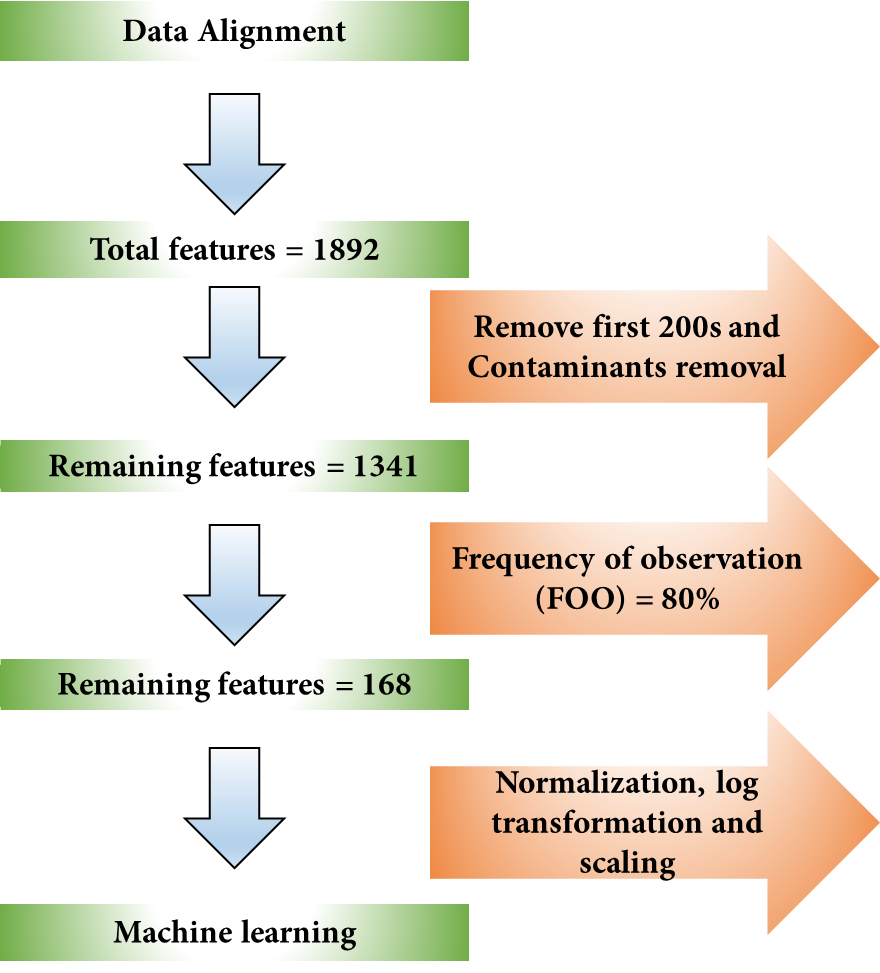
**

**Figure S7:** A flowchart of data analysis used in this study

**References:**

1. R Core Team. R: A Language and Environment for Statistical Computing. 2019.

2. Kuhn M. Building predictive models in R using the caret package. *Journal of Statistical Software.* 2008;28(5):1-26.

3. Kassambara H. ggpubr: ‘ggplot2’ Based Publication Ready Plots. 2020.

4. Wickham H. *ggplot2: Elegant Graphics for Data Analysis.* New York: Springer; 2016.

5. Thévenot EA, Roux A, Xu Y, Ezan E, Junot C. Analysis of the Human Adult Urinary Metabolome Variations with Age, Body Mass Index, and Gender by Implementing a Comprehensive Workflow for Univariate and OPLS Statistical Analyses. *Journal of Proteome Research.* 2015;14(8):3322-3335.
